# HPV-Based Screening, Triage, Treatment, and Followup Strategies in the Management of Cervical Intraepithelial Neoplasia

**DOI:** 10.1155/2013/912780

**Published:** 2013-04-14

**Authors:** Oscar Peralta-Zaragoza, Jessica Deas, Claudia Gómez-Cerón, Wendy Argelia García-Suastegui, Geny del Socorro Fierros-Zárate, Nadia Judith Jacobo-Herrera

**Affiliations:** ^1^Direction of Chronic Infections and Cancer, Research Center in Infection Diseases, National Institute of Public Health, Avenida Universidad 655, Cerrada Los Pinos y Caminera, Colonia Santa María Ahuacatitlán, Cuernavaca, 62100 Morelos, MEX, Mexico; ^2^National Institute of Medical Sciences and Nutrition “Salvador Zubirán,” Vasco de Quiroga 15, Tlalpan, 14000 México, DF, Mexico

## Abstract

Cervical cancer is the second most common cause of death from cancer in women worldwide, and the development of new diagnostic, prognostic, and treatment strategies merits special attention. Many efforts have been made to design new drugs and develop immunotherapy and gene therapy strategies to treat cervical cancer. HPV genotyping has potentially valuable applications in triage of low-grade abnormal cervical cytology, assessment of prognosis and followup of cervical intraepithelial neoplasia, and in treatment strategies for invasive cervical cancer. It is known that during the development of cervical cancer associated with HPV infection, a cascade of abnormal events is induced, including disruption of cellular cycle control, alteration of gene expression, and deregulation of microRNA expression. Thus, the identification and subsequent functional evaluation of host proteins associated with HPV E6 and E7 oncoproteins may provide useful information in understanding cervical carcinogenesis, identifying cervical cancer molecular markers, and developing specific targeting strategies against tumor cells. Therefore, in this paper, we discuss the main diagnostic methods, management strategies, and followup of HPV-associated cervical lesions and review clinical trials applying gene therapy strategies against the development of cervical cancer.

## 1. Introduction

Currently, 500,000 new cases of cervical cancer are diagnosed and 280,000 deaths occur each year worldwide, making cervical cancer the second most common malignancy affecting women worldwide [1]. The highest incidences occur in developing countries, where cervical cancer is the leading cause of cancer mortality in women. Clinical, epidemiological, and molecular data associate high-risk HPV infection with cervical cancer development [1]. The high-risk HPVs infect the basal epithelial cells of the cervix, and the subsequent expression of viral gene products is carefully regulated. During the cervical carcinogenesis process, HPV E6 and E7 viral oncogenes are expressed at low levels in proliferating basal cells, but transcription is activated as cells enter into the terminal differentiation pathway. For instance, E6 oncoprotein alters cell differentiation while E7 oncoprotein reactivates cellular proliferation, and these two viral oncoproteins together stimulate cell cycle progression. Thus, E6 and E7 oncogenes are retained and expressed in most cervical cancers, and sustained expression is required to retain the malignant phenotype [2, 3].

 During the last 50 years, screening programs based on conventional cytology have significantly reduced cervical cancer morbidity and mortality [4, 5]. Papanicolaou (Pap) smears are used as a primary screening method to detect precursor lesions or low-grade squamous intraepithelial lesions (LSILs). Detection of the early expression of HPV genes in combination with Pap smears may increase specificity in identifying LSIL associated with high-risk HPV infections and enable prevention of the development of precancerous lesions or progression to invasive cancer *in situ*. Molecular target screening should be able to detect HPV-like etiological agents as well as identify precancerous lesions. To detect HPV antigens and determine HPV prevalence in premalignant and precancerous lesions, and to assess the sensitivity and specificity of molecular target screening with respect to cytological, colposcopic, and anatomopathologic methods; immunohistochemical, molecular biology and microarrays techniques have been designed. Laboratory tests can detect HPV DNA and identify HPV genotypes; there are currently several molecular biology techniques used to detect HPV and identify the virus type with which the patient is infected. In summary, HPV nucleic acid can be detected either in cervical smears or biopsy specimens by various methods complementary to traditional cytology.

 Once the disease has spread beyond the confines of a radiotherapy, chemotherapy, or surgical field, no standard treatment is currently available. However, new molecular targeted drugs being tested in clinical trials might inhibit tumor progression and increase apoptosis, resulting in tumor response or stabilization; furthermore, gene therapy strategies could also be employed for the control of HPV-associated precancerous cervical lesions. In this paper, we discuss the role of HPV in cervical cell transformation and the main methods for the detection of high-risk HPVs, with special attention to application of HPV testing in screening, treatment, and followup of precancerous cervical intraepithelial neoplasia. We also examine the major gene therapy strategies against cervical cancer development that have been tested or are being tested in clinical trials.

## 2. The Natural History of Cervical Lesions

### 2.1. HPV Genome Structure and Functionality

 Currently, over 120 different types of HPV have been identified based on the genotype [6]. The HPVs are small DNA tumor viruses that replicate in differentiating epithelial cells of the epidermis and anogenital tract. The gene expression mechanisms of the HPV genome have been studied mainly in the HPV6, 11, 16, 18, and 31. Some common mechanisms involved in the regulation of their transcription are the presence of a specific promoter structure, a transcriptional enhancer specific for epithelial cells, regulation by progesterone and glucocorticoid hormones, silencers that apparently repress transcription in the basal layer of infected epithelia, nucleosome mediators of enhancer and silencer factors, nuclear matrix attachment regions that repress or stimulate transcription, and late promoters positioned very remote from the late genes [7].

 The early region (“E”) of the HPV genomes covers more than 50% of the virus genome and contains the E1, E2, E4, E5, E6, and E7 genes that are carefully expressed at the early and late stages of infection. The early proteins E1 and E2 are involved in viral DNA replication and viral RNA transcription, E4 is involved in cytoskeleton reorganization and E5, E6, and E7 are responsible for cellular transformation, and immortalization [8]. It has recently been demonstrated, using an E1-defective mutant of HPV16, that E1 protein is dispensable for maintenance replication and essential for initial and productive replication of HPV16 [9]. The E2 protein is a transcription factor that acts as an activator or repressor of the transcriptional activity of all HPV genes, and it has been observed that this protein is also involved in viral DNA replication via an interaction with protein E1 and episome maintenance [10, 11]. Therefore, E2 is a regulatory protein relevant for the establishment of infection and vital for the virus to complete the life cycle. On the other hand, the disruption of the E2 gene has been associated with malignant progression of HPV positive cervical cancer [12]. In particular, E2 proteins of HPVs and BPVs bind specifically to palindromic sequences ACCGN_4_CGGT that are concentrated within the viral LCR, where they regulate viral oncogene transcription. It has been demonstrated that E2 protein associates *in vitro* with several cellular transcription associate factors (TAFs), and these events function to activate HPV promoter over a relatively large distance in the viral genome [13]. Once the viral DNA has been integrated into the host cell genome, the E2 gene is disrupted or inactivated, and this event leads to a deregulation of HPV E6 and E7 oncogenes. 

 HPV16 E1^∧^E4 are products of a spliced transcript of the E1 and E4 open reading frames that can differ in length and/or degree of phosphorylation; some of these isoforms can associate with cellular keratin networks, leading to network disruption [14]. Recently, it has been shown that the cellular cysteine protease calpain cleaves the HPV16 E1^*∧*^E4 protein to generate species that lack the N-terminal fragment, and C-terminal fragments are able to multimerize to form amyloid-like fibers that can lead to accumulation of E1^*∧*^E4 and disruption of the normal dynamic of the keratin networks [15]. In addition, E1^*∧*^E4 might serve as scaffold, transport, or structural proteins, and E1^*∧*^E4 gene viral mutants do not support high-level replication of the viral DNA, suggesting that E1^*∧*^E4 is involved in HPV DNA replication [15].

 HPV E5 oncoprotein interacts with cellular host proteins, and these interactions are important for HPV's biological activity in cell transformation and evasion of the host immune response. Recent studies have highlighted the important role of E5 in cell transformation, tumorigenesis, and immune modulation, thus implicating E5 in pivotal steps of carcinogenesis [16]. For instance, high-risk HPV E5 can induce morphological and chromosomal changes in infected cells that can transform them into cancerous cells with increased nuclear size, increased DNA content, and tetraploidy [16]. Protein interaction studies have demonstrated that the first transmembrane domain of E5 from HPV16 and 31 interacts directly with the heavy chain component of the MHC class I through the leucine pairs present in this region; the transmembrane domain also interacts with Bap31, a chaperone of MHC class I [17]. HPV E5 contributes to a successful infection by inducing loss of surface MHC class I expression in the infected basal cells, preventing presentation of viral antigens to effector T-cells and thus, along with other mechanisms, such as prevention of inflammation, contributes to evasion of immune surveillance. Expression of E5 in the basal or suprabasal layers of the epithelium would lead to sustained cell proliferation to favor virus-infected cells, while extinction of its expression in the more superficial layers would permit cell differentiation and virion production. If E5 expression proceeds beyond early lesional stages, keratinocyte differentiation and immunological removal of infected cells do not take place and the lesion is at greater risk for neoplastic progression [16]. In primary human keratinocytes, the expression of E5 oncoprotein from HPV16 stimulates cellular DNA synthesis and, in cooperation with E7 oncoprotein, induces proliferation of primary cells [18]. In NIH 3T3 cells transformed with the E5 gene of rhesus papillomavirus, the phosphatidylinositol-3′-kinase was upregulated, suggesting that E5 plays a role in regulation of signal transduction pathways [16, 19].

 An early event in HPV-associated carcinogenesis during HPV DNA integration is a global perturbation of cellular gene expression by the E6 and E7 oncoproteins. The high-risk HPV E6 oncoprotein contains approximately 150 aminoacids and has two zinc finger motives formed by four C-X-X-C motives that are required for E6 functions [20]. One of the main well-defined interaction targets of E6 from HPV16 and HPV18 is the tumor suppressor protein p53. The p53 transcription factor is the tumor suppressor gene that is most frequently inactivated in human cancers and is involved in the control of cell proliferation and the response to genotoxic stress and DNA damage [21]. The inactivation of p53 by high-risk HPV E6 alters several cell processes including apoptosis, cell cycle arrest, cellular senescence, and differentiation. p53 inactivation occurs through the recruitment of E6AP protein, which is a ligase of the ubiquitin proteolysis pathway [20]. The E6-E6AP complex recognizes the p53 tumor suppressor protein, facilitates its polyubiquitination, and induces its rapid cleavage by the proteosome pathway [20]. The loss of p53 leads to an increase in the cell's genomic instability. High-risk HPV E7 oncoprotein, also involved in cellular transformation and immortalization, contains approximately 100 aminoacids and has three conserved regions called CR1, CR2, and CR3, which are critical for viral oncogenic activities. E7 oncoproteins, from low-risk as well as high-risk HPVs, may interact with the pRb tumor suppressor protein; however, high-risk HPV E7 has a greater affinity for pRb than does low-risk HPV E7 [20, 22]. Initially, interactions take place between E7 and the hypophosphorylated form of pRb, which induces the liberation of transcription factors from the E2F family, thus stimulating the expression of multiple genes involved in the progression of the cell cycle. These interactions influence the expression of genes involved in progression to S-phase of the cell cycle, such as cyclin A, cyclin D, and cyclin E. Furthermore, since pRb family members act as negative regulators of p16INK4A expression, the inactivation of pRb by HPV E7 oncoprotein results in upregulation of p16 cellular protein [20]. Other important interactions of E6 and E7 oncoproteins with cellular proteins such as AP-1, Bak, c-myc, Epoc-1, E6BP/ERC55, hAda3, IGFBP-3, Mi2, MPP2, NuMA, PDZ domain proteins, pRb, p21^waf1/cip1^, p27^kip1^, p53, p300/CBP, p600, TBP, Tyk2, and hTERT have been reported [20, 23]. E6 and E7 together exert effects on cell cycle control, as well as evasion of cell growth regulation, resistance to apoptosis, immune response escape, and angiogenesis-associated processes; in combination, these HPV oncoproteins efficiently immortalize and transform human keratinocytes to cause the malignant phenotype.

 Two new proteins codified in the early gene region have been recently identified, E3 and E8, which are present only in a few papillomavirus types (HPV 1, 11, 16, 31, and 33). A fusion protein, E8^*∧*^E2C, functions as a negative regulator for HPV DNA replication and plays a role in the control of viral copy number as well as in the stable maintenance of HPV episomes [24].

 The late region (“L”) of all HPV genomes, comprising almost 40% of the virus genome, is located downstream of the early region and encodes L1 and L2 ORFs for translation of a major (L1) and a minor (L2) capsid protein. Finally, the long control region (LCR), a segment of about 850 bp (*≈*10% of the HPV genome), has no protein-coding function but contains the origin of replication as well as multiple transcription factor binding sites important in the regulation of RNA polymerase II-initiated transcription from viral early and late promoters. HPV L1 capsid proteins in HPV-positive LSIL and HSIL are a major target of the cellular immune response in CIN [25]. Immunohistochemical testing, using antibodies against HPV structural protein L1, can confirm the presence of high-risk HPV L1 protein in abnormal epithelial cells observed in cytology or in squamous cell carcinomas [26].  Table 1 summarizes the main functions of HPV proteins.

### 2.2. HPV and the Natural History of Cervical Cancer

 There is a close relationship between high-risk HPV infection and cervical cancer development. However, it is well documented that HPV is a very common sexually transmitted virus, and most infections do not cause cervical intraepithelial neoplasia. Epidemiological studies have reported HPV prevalence between 10% and 40% in women with no cytological abnormalities [27]. Moreover, data suggest that a large part of the population may at some point in their lives have an HPV subclinical infection, particularly while young since older women may develop acquired immunity to HPV; the likelihood of HPV infection increases with risk factors such as high alcohol consumption and more sexual partners [27, 28]. This evidence suggests that while HPV infection is a relevant event in the process of cervical carcinogenesis, other factors participate in the development of a malignant phenotype.

 The process by which cervical cancer develops is complex, and a combination of environmental, viral, and host factors together increases the risk of progression. Three main HPV-liked molecular events have been identified as triggers of carcinogenesis: (a) viral DNA integration in the host genome; (b) expression of viral proteins (E1, E2, E3, E4, E5, E6, and E7); (c) interactions between E2, E6/E7, and cellular proteins. In general, HPV epithelial infections occur either through mechanical microabrasions or infection of the area, where basal cells undergo a transformation from a columnar to a squamous epithelium. When basal cells are infected, they migrate to the lumen and express the capsid genes L1 and L2. In subclinical infections of low-grade lesions, the viral genome is then replicated as an episome and encapsidated into the nuclei of cells in the upper layer epithelium. Thus, protected viral particles are able to infect new areas of epithelium or be sexually transmitted. In some cases, the infection progresses to high-grade lesions and cervical carcinoma, a process associated with the integration of the HPV genome into the host genome due in part to the loss of the transcriptional repression exerted by the E2 protein. 

Invasive cervical carcinoma is preceded by precursor lesions, which are characterized by disturbances of cellular maturation and stratification, as well as atypical nuclei, which tend to promote cellular immortalization and transformation [2]. The time-specific expression of viral oncogenes enables HPV to integrate into the cellular genome during the division of basal cells and the differentiation of basal epithelium to stratified epithelium [3]. Prior to integration in the host cell genome, episomal HPV expresses the genes E1 and E2 to suppress expression of the oncogenes E6 and E7, allowing the virus to invade various cells and evade detection by the immune system [29]. When the HPV viral genome integrates into the cellular genome, expression of E2 is disrupted and expression of E6 and E7 predominates. When HPV infection occurs, HPV DNA can exist in one of two alternative molecular conformations: episomal or integrated. When the viral DNA is inside of the nucleus but not bound to host DNA, it is in the episomal conformation, which is characteristic of low-grade lesions. When HPV DNA binds to host cellular DNA, it is considered to be in the integrated conformation, which is found in high-grade lesions and invasive carcinomas [30, 31]. The physical state (extrachromosomal or integrated) of HPV DNA has been evaluated via analysis of the expression of HPV E6 and E7 mRNA transcripts. All HPV types have been found to be extrachromosomal in benign cervical smears, CIN 1 and CIN 2. HPV16 existed in all physical states in CIN 3 and carcinoma *in situ*, whereas HPV18 was found only in mixed and integrated forms in these stages of cervical carcinogenesis. HPVs 31, 33, 52b, and 58 appeared most commonly in the extrachromosomal form in all stages of lesions, while the integrated form of these HPV types was present only in CIN 3 and in carcinoma *in situ* [32].

## 3. Application of HPV Testing in Screening, Treatment, and Followup of Precancerous Cervical Intraepithelial Neoplasia

### 3.1. HPV Testing in Cervical Cancer Screening

 Recommendations for cervical cancer screening released in 2012 by the US Preventive Services Task Force (USPSTF) and separately by a partnership among the American Cancer Society/American Society for Colposcopy and Cervical Pathology/American Society for Clinical Pathology (ACS/ASCCP/ASCP) no longer indicate annual screening with cervical cytology but rather screening at three- to five-year intervals with cytology and optional incorporation of testing for high-risk HPV infection. For women aged 21–29, the guidelines indicate screening with cytology (i.e., the Papanicolaou test) every three years; HPV testing is not recommended for this age group because younger women are more likely to experience transient infections and low-grade lesions that do not progress to precancerous lesions and do not require identification or treatment. Women older than 65 with evidence of adequate negative prior screening and no history of CIN2+ within the last 20 years should not be screened. Screening should not be resumed for any reason, even if a woman reports having a new sexual partner. For women aged 30–65, however, both sets of guidelines indicate screening with a combination of cytology and HPV testing every five years, the “preferred” method, or screening with cervical cytology alone every three years (“acceptable”) [33]. Despite its success in reducing rates of cervical cancer in the developed world, conventional cytology has its limitations; a meta-analysis showed that it has an average sensitivity of 51% and a specificity of 98% compared with colposcopy or histological analysis of biopsies [34]. According to a 2003 study, one third of false-negative diagnoses can be attributed to errors in the interpretation of the film used to identify cellular abnormalities and the other two thirds to the quality of the preparation of that film [35]. The revised national recommendations are based on a number of studies showing improved performance of coscreening over cytology alone, though the USPSTF and ACS/ASCCP/ASCP do not go as far as many of these studies in suggesting HPV testing as a stand-alone screening method. 

 The US Food and Drug Administration (FDA) currently approves three HPV DNA tests for clinical use: Hybrid Capture 2, manufactured by Qiagen; Cervista HPV HR (Hologic); the cobas HPV test (Roche Molecular Systems). The most widely used test is Hybrid Capture 2 (HC2), a second-generation commercial test designed to detect HPV types classified as high-risk. HC2 is a nucleic acid hybridization assay that utilizes an HPV RNA probe mixture containing probes for 13 high-risk HPVs. Samples containing HPV DNA hybridize with the RNA probe and are captured on a plate coated with antibodies conjugated to alkaline phosphatase; when alkaline phosphatase's substrate is added, light is emitted and measured via luminometer. The test's limitations include possible cross-hybridization of low-risk with high-risk HPV types as well as possible false positives in contiguous samples due to chemiluminescent emission of high-load HPV samples [36]. A 2003 study found that the sensitivity of the HC2 test alone was lower than either the conventional Pap smear or liquid-based smear, in the contexts of systematic screening and triage of abnormal squamous cells of unidentified significance (ASC-US) [36]. The authors' recommendation not to use HC2 HPV testing in the management of patients with ASC-US is not concordant with the conclusions of meta-analyses by Arbyn et al. [37] or Cuzick et al. [38], perhaps because the de Cremoux et al. study [36] assumes optimized readings of conventional cytologic techniques. Nevertheless, the questions raised about the sensitivity and specificity of HC2 have encouraged research and development of alternative tests. A major limitation of HC2 is that it cannot determine specific high-risk HPV type, the identification of which may help clinicians provide prognosis and appropriate treatment and followup, depending on the strength of the infecting strain's association with cancer and disease recurrence.

 An established method for the detection of HPV DNA is PCR amplification with primers derived from consensus sequences of either the conserved E1 or L1 open reading frames of the viral genome. The primer sets MY09/11 and GP5+/GP6+ are both able to amplify a wide range of HPV types. The MY09/11 primers are synthesized with several degenerate nucleotides and constitute a mixture of 25 primers, while the GP5+/GP6+ primers have fixed nucleotide sequences but use a lower annealing temperature during PCR to detect more HPV types [39]. Two-step or “nested” PCR using both primer sets (MY09/GP5+/GP6+/MY11) has been shown to be a highly sensitive and reliable method for the detection of HPV infection [40]; however, it is not practical for routine diagnosis due to the time required and susceptibility to contamination. The MY09/11 primers were redesigned as general, no-degenerate primers, PGMY09/11, to improve the sensitivity, specificity, and reproducibility of one-step MY-PCR. An analysis in 2004 found that compared to MY-PCR, PGMY-PCR is slightly more sensitive but significantly more efficient and has a larger HPV detection range, detecting the MY-PCR-negative types HPV-42, HPV44, HPV51, HPV87, and HPV89 [41]. SPFs are primers developed for universal detection of HPV which target a highly conserved region of the HPV L1 gene, amplifying numerous genital HPV types in one reaction [42]. Real-time quantitative PCR (qPCR) methods can also be used to analyze the expression of HPV early genes in LSIL and HSIL. qPCR assays produce linear results over a wide range of target concentrations. Quantitation over a range of 101 to 106 initial HPV copies was achieved using qPCR detection of the accumulation of fluorescence with cycle number [43]. By detecting and quantifying high-risk HPV, qPCR demonstrated a relationship between viral DNA quantity and risk of cervical carcinoma for the HPV types most commonly found in cervical tumors [44]. Following PCR, the HPV type may be identified through several methods including direct cycle sequencing, which has long been considered the optimal approach for accurate HPV identification and genotyping [45]. A novel method, the HPV INNO-LiPA (Innogenetics NV, Ghent, Belgium), a reverse hybridization line probe assay, was found to have a higher degree of sensitivity, be more rapid, and be more capable of identifying multiple infections compared to PCR sequencing, though PCR sequencing allowed for the detection of a broader range of HPV types [46].

 Baleriola et al. [47] has evaluated the efficacy of a new HPV diagnostic kit, the high-risk HPV detection kit, manufactured by Human Genetic Signatures (Australia). The kit treats a Pap smear and/or liquid-based cytology sample to isolate DNA and simplify the genome from four bases to three by converting cytosine to uracil and eventually thymine. Simplification of the genome allows for the design of high-risk PCR primers that contain fewer mismatches and have less cross-reactivity. PCR product is detected with agarose gel electrophoresis and subsequent reflex PCR can identify the individual strain(s). The HGS high-risk HPV detection kit was found to have a statistically significant higher positive predictive value as well as specificity, and a statistically significant lower rate of false positives compared with HC2.

 Another detection method, the HPV oligonucleotide microarray (commercially available from Biomedlab Co., Republic of Korea), was evaluated by An et al. [48] and Kim et al. [49]. The HPV-DNA-Chip contains 22 type-specific probes, 15 of high-risk group (16, 18, 31, 33, 35, 39, 45, 51, 52, 56, 58, 59, 66, 68, and 69) and 7 of low-risk group (6, 11, 34, 40, 42, 43, and 44) that allow detection of the generic and type-specific sequence of HPV types. DNA isolated from patient specimens is amplified via PCR and labeled with Cy5-dUTP, then hybridized onto the Chip and washed, followed by visualization of hybridized signals with a DNA Chip Scanner. While the time required for sample processing is roughly equal to the HC2 assay, the HPV-DNA-Chip method allows for the identification of individual HPV type as well as detection of multiple HPV infections, which occurred in 21 of 140 cervical samples in the Kim et al. study [49]. When the HPV oligonucleotide microarray was added to normal cytologic screening, sensitivity improved significantly; An found that of 33 patients who were underdiagnosed using the cytologic smear method, 30 were identified as HPV positive by the HPV DNA Chip test [48].

### 3.2. Sensitivity and Specificity of HPV Testing

 The application of HPV DNA testing alone or in combination with cervical cytology in screening, treatment, and followup of cervical cancer has been the focus a great deal of research in the past two decades. A 2006 meta-analysis of split-sample studies in Europe and North America that compared HPV testing with routine cytology found that HPV testing had significantly higher sensitivity than cytology, while cytology had a higher positive predictive value (PPV), in detecting a cervical intraepithelial lesion of grade II or worse (CIN2+), confirmed via subsequent histologic examination [38]. One study found that the lower specificity of HPV testing could be overcome by performing cytology triage, that is, referring high-risk HPV-positive patients to colposcopy and/or biopsy only when subsequent cytology was suggestive of dysplasia or cancer [50]. The ATHENA (Addressing the Need for Advanced HPV Diagnostics) clinical trial similarly found that HPV testing was more sensitive but less specific than cytology for detection of cervical intraepithelial lesion of grade III or worse (CIN3+); based on their results, the authors recommend use of HPV testing as a primary screening test and use of a specific test such as liquid-based cytology to refer women for colposcopy [51]. The ATHENA study utilized the cobas HPV test, a fully automated test with the ability to detect HPV16 individually, HPV18 individually, and a pool of 12 other high- or intermediate-risk HPV genotypes (31, 33, 35, 39, 45, 51, 52, 56, 58, 59, 66, and 68). This feature of the test allowed for individual genotyping for HPV16 or HPV18, high-risk HPVs associated with around 70% of invasive carcinomas [52]. Triage to immediate colposcopy based on detection of either or both of these HPVs provided increased and more reliable identification of women with CIN3+ than triage based on detection of ASC-US or worse with liquid-based cytology [51]. A separate potential use of HPV testing is as a triage method for women who have received equivocal results from cytological testing. A 2004 meta-analysis of virologic and cytologic screening studies found that for patients with ASC-US identified by Pap smear, HPV-DNA testing with the HC2 assay had higher sensitivity than repeat cytology for detecting CIN2+, though the specificity of both methods was low [37].

### 3.3. Effect of HPV Testing on Incidence of CIN and Cervical Cancer

 Several recently published studies have examined long-term outcomes of screening programs that incorporate HPV testing. A large Netherlands-based randomized controlled trial, the Population-Based Screening Study Amsterdam (POBASCAM), determined that women who were screened for HPV DNA (with the GP5+/6+ PCR method) in combination with cytology had fewer cervical intraepithelial neoplasias of grade 3 (CIN3) and fewer cervical carcinomas in a subsequent screen five years later, than those who were screened with cytology alone [53]. The protective effect of high-risk HPV testing was attributable in large part to early detection and treatment of high-grade cervical lesions caused by HPV16. A separate large randomized controlled trial in Italy also found a significant decrease in cases of invasive cancer detected in women screened with HPV testing alone or in combination with cytology compared to women screened with cytology alone. The authors conclude that their data support the use of stand-alone HPV testing as the primary screening test [54]. In a cohort study of cervical cancer incidence among coscreened patients at Kaiser Permanente Northern California, a large managed care organization, a negative HPV test result was sufficient to ensure a patient of an extremely low risk of CIN3 or cervical cancer (3.2/100,000 women/year) over a five-year testing interval [55]. Similar to the results of the Italian trial [54], the study found that a negative cytology result provided no extra reassurance against cancer.

### 3.4. Application of HPV Testing in Low-Resource Settings

 High-risk-HPV testing may be particularly valuable in low-resource settings, where the majority (80%) of cervical cancer cases occur, and where the infrastructure does not exist to implement effective cytology-based screening programs. In Colombia, for example, guidelines issued by the National Cancer Institute recommend screening based on a 1-1-3 cytology strategy (annual conventional cytology until two consecutive negative smears and every three years thereafter); however, coverage rates are low, with one-year coverage of women aged 25–69 ranging from 43.3% in the state of Boyacá to 56.8% in Caldas and three-year year coverage ranging from 66.6% in Magdalena to 81.3% in Caldas [56]. A comparative analysis of HPV-DNA testing at three- or five-year intervals versus conventional cytology at 1-1-3 or 1-1-1-3 intervals found that HPV-DNA testing at a five-year interval was the least costly per year of life saved (USD$44/YLS). While five-year HPV-DNA testing had a lower effectiveness than the other strategies, there was not a substantial difference in years of life saved; furthermore, the longer time interval may allow for screening to reach more women. This strategy was cost-effective when cost per test was less than or equal to US$31; although actual costs per test are currently much higher, implementation of the technology in a national screening program would lower costs to the rate assumed in the study [57]. In South Africa, where current public health guidelines recommend screening with conventional cytology (Pap smear) at a 10-year interval, HPV-based screening was found to reduce cervical cancer incidence and mortality by 41% and 47%, respectively, relative to conventional screening [58]. While the study found that HPV testing was more expensive than conventional cytology, analysis of the incremental cost-effectiveness ratio (the ratio of the difference in costs to the difference in effectiveness between two alternative screening strategies) determined that HPV-based screening would be a cost-effective option in South Africa.

 The technical requirements of most HPV-DNA tests, however, make them impractical for use in low-resource settings. A new HPV test designed specifically for use in developing countries was assessed for clinical accuracy in screening tests in county hospitals in rural China [59]. The careHPV test (Qiagen), a signal-amplification assay that detects target HPV-DNA from 14 HR-HPV types (16, 18, 31, 33, 35, 39, 45, 51, 52, 56, 58, 59, 66, and 68), is broadly based on the HC2 test with modifications specific to low-resource settings, such as a reduced assay time (2.5 h or less, compared with up to 6 h for HC2), which allows for same-day testing and clinical followup; in addition, the test uses only a small footprint of bench-top work space and requires no main electricity or running water. The test was found to be highly accurate for the detection of CIN2+, with no significant difference between the sensitivity and specificity of the careHPV test compared to the HC2 test on cervical specimens, which suggests that it may be a feasible and effective approach for implementing HPV-based screening in developing regions of the world.

### 3.5. Management of Cervical Preinvasive Lesions

 Following histologic identification of cervical intraepithelial neoplasia, surgical treatment is recommended for preinvasive lesions of grade CIN2 or CIN3. The 2006 Consensus Guidelines published by American Society for Colposcopy and Cervical Pathology (ASCCP) and its partner organizations utilize a two-tiered system in which histologically diagnosed CIN1 is classified as a low-grade lesion and CIN2/3 are classified as high-grade precursors [60]. No treatment is recommended for CIN1 at the time of diagnosis because most low-grade lesions regress spontaneously; studies have reported spontaneous clearance rates that reach 91% within 24–36 months of a cytological result of LSIL [61, 62]. Instead, followup with HPV DNA testing every 12 months or repeat cervical cytology every 6 to 12 months is indicated; if CIN1 persists for at least two years, either continued observation or treatment is acceptable. HPV genotyping may be of predictive value in identifying women whose CIN1 are most likely to progress to high-grade lesions and/or cancer, since HPV16 and HPV18 are associated with a substantially higher risk of progression. An Oregon-based cohort study reported that 10-year cumulative incidence rates of ≥CIN3 were 17% among HPV16+ women and 14% among HPV18+ (HPV16−) women, but only 3% among HR-HPV+ women negative for HPV16 or HPV18, and less than 1% among non-high-risk HPV- infected women [63].

 Although the risk of CIN2 progressing to invasive cancer is low, diagnosis of CIN2 has limited reproducibility and validity, and thus CIN2 is used as the treatment threshold for safety reasons [64]. Surgical treatment options for CIN2/3 include ablative methods that destroy the affected cervical tissue *in vivo*, such as cryotherapy, laser ablation, electrofulguration, and cold coagulation, as well as and excisional methods that remove the affected tissue and provide a specimen for pathological examination; these include cold-knife conization, laser conization, electrosurgical needle conization, and loop electrosurgical excision procedures (LEEPs), also known as large loop excision of the transformation zone (LLETZ). LEEP is now the favored procedure worldwide; although it has not been shown to be more effective than other methods, it has clinical advantages such as less operating time and lower rates of hemorrhage, pain, and infection, and it is less expensive and technically simpler than laser treatment [65]. All of the excisional methods of treatment are associated with adverse effects on future pregnancies including increased risk of low birth weight and premature rupture of membranes [66]; however, only cold-knife conization is consistently associated with serious adverse outcomes such as perinatal mortality and severe preterm delivery [67]. Ablative treatment methods typically destroy a smaller amount of cervical tissue and thus are less frequently linked to obstetric morbidity, but ablation may not be appropriate where there is suspicion of invasion, a larger affected area, or transformation zones extending deep in the endocervical area [67].

### 3.6. Followup of Cervical Intraepithelial Neoplasia: The Role of HPV Testing

 Postsurgical followup of women treated for CIN 2/3 with ablative or excisional methods is critical, since treatment failure rates are estimated at 5%–15% across all treatment modalities [68]. Failure can take the form of residual or recurrent CIN or invasive cervical cancer. One study reported that in the first 6 years after treatment, recurrence rates of CIN2/3 were 14.0% for women originally treated for CIN 3 and 9.3% for CIN 2, while incidence of invasive cancer was 37 per 100,000 woman-years in the cohort treated for CIN compared to 6 per 100,000 woman-years in the comparison cohort [69]. The ASCCP guidelines indicate followup with HPV DNA testing at 6–12 months or with cytology at 6-month intervals, with colposcopy recommended for women who are HPV-positive or have a repeat cytology result of ASC-US or worse [60]. The sensitivity of HPV-DNA testing in detecting treatment failure is high, reported in one meta-analysis to reach at least 90% at 6 months after treatment, compared to 65% for conventional cytology, while the specificity of HPV testing ranged from 44% to 95% in the studies analyzed [70]. A British study reported that at 6 months after treatment, combined analysis of cytology and a single high-risk HPV test predicted high grade disease recurrence with a 100% sensitivity, 92% specificity, 100% negative predictive value (NPV), and 20% positive predictive value (PPV) [71]. In the case of persistent HPV infection after treatment, HPV genotyping may allow for the classification of women into risk groups. Venturoli found that women with persistent HPV16 and/or HPV18 infection at 6 months after treatment had a higher recurrence rate (82.4%) of CIN of any grade than HPV16/18-negative women with persistence of at least one other high-risk HPV type (66.7%) or at least one probably high-risk type (HPV39, 51, 56, 59, 68, 26, 53, 66, 73, and 82) (14.3%) [72]. It is highly recommendable, therefore, that women with persistent high-risk HPV and particularly those with HPV16/18 infection at 6 months followup undergo colposcopy, per the ASCCP guidelines. In summary, high-risk HPV testing appears to be a very sensitive method for detecting recurrence of low- or high-grade CIN following surgical treatment for CIN2/3, and its near-perfect negative predictive value allows women who have cleared their HPV infection to be returned to routine screening.

## 4. Gene Therapy Clinical Trials for Cervical Cancer

 If lesions are not identified and treated at the precancerous stages, treatment options are limited for women with metastatic or recurrent cervical cancer. Unfortunately, only up to one third of patients with metastatic and recurrent disease will respond to drug chemotherapy, and these responses are short-lived, on the order of months. Gene therapy strategies targeted to HPV products could represent new treatment options for cervical cancer and also other tumors in which HPV participates as a cancer promoter. Currently, it is well known that HPV E6 and E7 interact with a plethora of cellular proteins, both nuclear and cytoplasmic, and participate in molecular pathways involved in the activation and establishment of the tumor phenotype. Hence, it is feasible to design experimental strategies aimed to block the expression of viral oncoproteins. HPV E6 and E7 oncoproteins are excellent candidates for HPV therapeutic vaccination strategies, and the most of the therapeutic vaccines that have been tested in preclinical and clinical trials focus on interacting with antigen-presenting cells to stimulate cytokine production and T-cell activation. In addition, several efforts are focusing on the development HPV therapeutic vaccines based in attenuated virus and bacterial vectors, HPV peptide and proteins, and specific tumor and dendritic cells, as well as naked plasmid DNA-based vaccines.

Table 2 summarizes in systematic format information about active cervical cancer gene therapy clinical trials worldwide from 1989 to 2012. The data were compiled from official agency sources such as the RAC, GTAC, and OBA/RAC websites [73]. In many clinical trials, the safety, tolerability, and immunogenicity of HPV E6 and E7 oncogenes vaccines are evaluated in combination with immunotherapeutic and chemotherapeutic drugs. There are several active studies and recruiting participants, for example, in trial US-0595 evaluates the side effects and best dose of pNGVL4a-Sig/E7(detox)/HSP70 which is an antigen-specific DNA-vaccine consisting of the coding sequence of a signal peptide (pNGVL4a-Sig), a detox of HPV16 E7, and the heat shock protein 70 (HSP70), in patients with CIN 2/3. Trial US-0928 proposes to study the side effects and the best dose of vaccine therapy, and to evaluate how well it works when given with or without imiquimod in treating patients with CIN 3. In trial US-0984, the efficacy and safety of different routes of administration of a naked plasmid DNA vaccine are analyzed in patients with HPV16+ CIN 2/3. Trial US-1082 analyzes the side effects of ADXS11-001, an HPV therapeutic vaccine containing a live-attenuated strain of *Listeria monocytogenes* encoding HPV16 E7 fused to a nonhemolytic listeriolysin O protein, in patients with persistent or recurrent cervical cancer. Trial US-1093 is a randomized, placebo-controlled study to determinate the safety and efficacy of the VGX-3100 DNA vaccine that contain plasmids targeting E6 and E7 proteins of both HPV16 and HPV18, which are delivered via electroporation to adult women with biopsy-proven HPV16 or HPV18-associated CIN 2 or 3 [73, 74]. It is important to note that proper clinical trial design is critical to ensure the scientific validity of results, the potential benefits that will accrue to society from the knowledge gained, and the ethics of conducting experimentation on human participants. Different design choices have implications for the risks and benefits to human participants in a trial and for the eventual applicability of research results in clinical practice. A classic approach in many trials is to compare an intervention of interest to standard treatment. Yet, whether and how to include such a comparison arm is a complex question in situations where multiple modalities are used, or where there is a lack of consensus in the professional community about which treatment is best.

 Although there is currently limited clinical information about gene therapy in cervical cancer patients, then trials discussed could establish proof of concept; therefore, it could be feasible to use gene therapy *in situ*. However, the choice of gene target is the most relevant part on this kind of clinical protocols. In summary, the clinical trial findings will address broad issues about gene therapy vaccines including efficacy, duration of protection, and global impact of vaccination on HPV-related tumors; though these vaccines have the potential to significantly improve cervical cancer outcomes, continued screening will still be required after intervention.

 On the other hand, although many studies have described the induction of cancer cell death* in vitro* by administration of specific small interference RNAs (siRNAs) for HPV16/18 E6 and E7 oncogenes in cervical cancer cells *in vitro*, few protocols have been complemented with animal tumor models with demonstrated eradication of tumors *in vivo* [75, 76]. This is a necessary phase in drug development before siRNA technology can be applied in clinical studies in humans. An aspect that needs to be analyzed in depth is the question of dosage quantities and the efficiency of siRNAs against a particular tumor. Although the first studies of siRNA against cervical cancer used chemically synthesized siRNAs, subsequent reports have used other molecular vectors to induce transcription of bioactive siRNAs with suppressive effects on tumor evolution both *in vitro* and *in vivo* [77–79]. When molecular vectors were used, the expression of the HPV oncogenes was inhibited in a more efficient manner. The specific delivery of siRNAs is still a limiting condition in the different models under study. However, the use of adhesive biogels, in combination with liposomes and chemotherapeutic drugs, shows promise, providing a greater efficiency in the release and dosage of siRNAs at the tumor site [80]. The relevance of silencing the expression of HPV E6 and E7 oncogenes with siRNAs will be better appreciated once such strategies are applied in clinical protocols. This goal will require adequate analysis and design of siRNA sequences to induce silencing of the E6-E7 bicistron, selection of appropriate of cloning vectors for siRNAs, and selection of molecular transport vehicles for siRNAs to protect them from the action of endonucleases and allow their administration in a site-specific and dose-dependent manner, as well as in the design of treatment schemes like chemotherapy, radiotherapy, or immunotherapy, to be used in combination with siRNAs. Though much progress remains to be made, siRNA technology represents a powerful gene therapy strategy against the development of cervical cancer.

## 5. Conclusions and Perspectives

 Although early stage cervical cancers have a good prognosis with a 5-year survival rate greater than 80%, clinical and epidemiological evidence suggests that the natural history of HPV in young women (aged <30 years) may be such that establishment of a high-grade CIN lesion occurs early in the course (within 2 years) of a high-risk HPV infection. The consequences of HPV infection will depend on the infecting HPV type and site of infection, as well as on host factors that regulate viral persistence, regression, and latency. HPV testing and identification of high-risk strains and multiple infections has potential applications in the screening, treatment, and followup of cervical intraepithelial lesion. The identification and subsequent functional evaluation of host proteins associated with HPV E6 and E7 oncoproteins is the major challenge for their utilization as molecular biomarkers and may provide useful information in understanding cervical carcinogenesis for development of specific targeting strategies against tumor cells. Many experimental HPV vaccine strategies are being developed and tested in preclinical and clinical trials, in combination with immunotherapy and chemotherapy approaches to control of HPV-associated cervical lesions and invasive cancers. The principal factor for the prevention and treatment of cervical cancer is the education of the society, which requires different strategies in developing and industrialized countries [81–84]. The costs of different screening, treatment, and followup strategies, as well as the economy and public health policies of the regions of their potential application, are relevant in providing effective and accessible care to the patient, while meeting family and government budgets. Consider that the total health care cost associated with the screening and treatment of cervical cancer in the US is estimated to be $6 billion dollars per year [85], which does not account for additional costs to the patient, the patient's family, and society (economic and social). 

 In conclusion, educational strategies and organized screening programs to detect HPV must be implemented alongside research and development of new therapeutic vaccines infection in order to reduce rates of HPV infection and cervical cancer. New gene therapy and siRNA-based approaches could represent a major step in reducing morbidity and mortality, but more work is required to achieve clinical efficacy at a level that can challenge current therapy.

## Figures and Tables

**Table 1:** Major functions of HPV proteins.

HPV proteins	Functions	Reference
E1	Involved in productive HPV replication.	[9]
E2	Regulates transcription from different HPV promoters.	[13]
E3	Recently identified gene located in early gene region and found only in a few papillomavirus types (HPV1, 11, 16, 31, 33); its function has not been identified.	[24]
E4	Represent up to 30% of total wart protein produce by HPV-1a and might serve as scaffold, transport, or structural protein.	[14]
E5	Inactivates with cellular host proteins (MHC I, Bap31); these interactions are important for the biological activity of the protein in cell transformation and evasion of the immune response. Play a role in regulation of transduction pathways through up-regulation of phosphatidylinositol-3′-kinase.	[16, 17, 19]
E6	Inactivates the tumor suppressor protein p53, involved in the control of cell proliferation and cell response to genotoxic stress and in DNA damage.	[20]
E7	Interacts with the pRb tumor suppressor protein. These interactions influence the gene expression involved in progression to S-phase of the cell cycle, such as cyclin A, cyclin D, and cyclin E genes.	[20, 22]
E8	Recently identified gene located in early gene region and found only in a few papillomavirus types (HPV 1, 11, 16, 31, 33). A fusion protein, E8^*∧*^E2C, functions as a negative regulator for HPV DNA replication playing a role in the control of viral copy number as well as in the stable maintenance of HPV episomes.	[24]
L1 L2	Major capsid protein. Minor capsid protein.	[26]

**Table 2:** Active gene therapy clinical trials for cervical cancer worldwide from 1989 to 2012.

ID trial	Trial title/country	Indication/clinical phase	Status/year approved-initiated	Gene(s) transferred	Vector used/administration route	Gene delivery
BE-0024	A randomized, double blind, placebo-controlled, parallel group, multicenter study of the safety and response rate of 3 subcutaneously administered doses of 5 × 10^7^ pfu RO5217790 in patients with high grade cervical intraepithelial neoplasia grade 2 or 3 associated with high risk HPV infection. Belgium	Cervical intraepithelial neoplasia. Phase I	Open 2009-ND	(i) delE6 (ii) delE7 (iii) IL-2	Vaccinia virus/ND	ND
CN-0010	Gendicine intratumoral injection combined with radiotherapy for advanced cervical carcinoma. China	Cervical carcinoma. Phase III	Open 2008-ND	p53	Adenovirus/intramuscular	ND
ES-0010	A randomized, double blind, placebo-controlled, parallel group, multicenter study of the safety and response rate of 3 subcutaneously administered doses of 5 × 10^7^ pfu RO5217790 in patients with high grade cervical intraepithelial neoplasia grade 2 or 3 associated with high risk HPV infection. Spain	Cervical intraepithelial neoplasia. Phase I	Open 2009-ND	(i) delE6 (ii) delE7 (iii) IL-2	Vaccinia virus/ND	ND
FR-0032	Phase II trial to assess efficacy of TG4001 (MVA-HPV-IL2) in patients with grade 2/3 cervical intra epithelial neoplasia (CIN 2/3) linked to HPV16 infection (protocol TH4001.07). France	CIN 2 and 3. Phase II	Open 2004-ND	(i) IL-2 (ii) HPV16	Vaccinia virus/ND	ND
MX-0001	Clinical protocol. A phase II study. Efficacy of the gene therapy of the MVA E2 recombinant virus in the treatment of precancerous lesions (NIC I and NIC II) associated with infection of oncogenic human papillomavirus. Mexico	Cervical cancer. Phase II	Open ND-ND	ND	Adenovirus/ND	ND
UK-0041	Use of a recombinant vaccinia vaccine (TA-HPV) to treat cervical intraepithelial neoplasia III. UK	Cervical intraepithelial neoplasia III. Phase I	Open 1996-ND	HPV E6 and E7 oncogenes	Poxvirus/ND	ND
UK-0042	Use of a recombinant vaccinia vaccine (TA-HPV) to treat cervical intraepithelial neoplasia III. UK	Cervical intraepithelial neoplasia III. Phase I	Open 1997-ND	HPV E6 and E7 oncogenes	Poxvirus/ND	ND
UK-0071	A phase II, multicenter, double-blind, placebo-controlled, dose finding study of ZYC101a in the treatment of high-grade squamous intraepithelial lesions of the uterine cervix. UK	Anogenital neoplasia III. Phase II	Open 2001-ND	HPV E6 and E7 oncogenes	Naked plasmid DNA/ND	ND
UK-0074	TA-HPV recombinant vaccinia virus expressing the human papillomavirus 16 and 18 E6 and E7 proteins: application to amend currently approved protocol to add a clinical trial involving prime-boost strategy of TA-CIN administered in association with TA-HPV in high grade anogenital intraepithelial neoplasia (AGIN) patients (PB-HPV/01). UK	Cervical cancer. Phase I	Open 2001-ND	HPV E6 and E7 oncogenes	Vaccinia virus/ND	ND
US-0592	A phase 1 study to determine the safety and immunogenicity of vaccination with Listeria monocytogenes expressing human papilloma virus type 16 E7 for the treatment of progressive, recurrent, and advanced squamous cell cancer of the cervix. USA	Cervical cancer. Phase I	Open 2003-ND	HPV E7 oncogene	Listeria monocytogenes/intravenous	*In vivo *
US-0595	A phase I/II clinical trial of pNGVL4a-Sig/E7 (detox)/HSP70 for the treatment of patients with HPV16+ cervical intraepithelial neoplasia 2/3 (CIN2/3). USA	Cervical cancer. Phases I and II	Open 2003-ND	HPV16 E7 oncogene	Naked plasmid DNA/intramuscular	*In vivo *
US-0916	Phase I, open-label, dose escalation study to evaluate the safety, tolerability, and immunogenicity of human papillomavirus (HPV) DNA plasmid (VGX-3100) + electroporation (EP) in adult females with histological diagnosis of grade 2 or 3 cervical intraepithelial neoplasia (CIN). USA	Cervical cancer. Phase I	Open 2008-ND	(i) HPV16 E6 and E7 oncogenes (ii) HPV18 E6 and E7 oncogenes	Naked plasmid DNA/intramuscular	*In vivo *
US-0928	A phase I efficacy and safety study of HPV16-specific therapeutic DNA-r vaccinia vaccination in combination with topical imiquimod in patients with HPV16+ high grade cervical dysplasia (CIN3). USA	HPV16+ high-grade cervical dysplasia. Phase I	Open 2008-ND	(i) HPV16 + HPV18 (ii) E6 + E7 oncogenes	Naked plasmid DNA + Vaccinia virus/intramuscular	*In vivo *
US-0958	A randomized, double blind, placebo-controlled, parallel group, multicenter study of the safety and response rate of 3 subcutaneously administered doses of 5 × 10^7^ pfu R05217790 in patients with high grade cervical intraepithelial neoplasia grade 2 or 3 associated with high risk HPV infection. USA	Cervical intraepithelial neoplasia (CIN). Phase II	Open 2008-ND	(i) HPV E6 and E7 oncogenes (ii) IL-2	Vaccinia virus/intramuscular	*In vivo *
US-0984	A pilot study of pNGVL4a-CRT/E7(detox) for the treatment of patients with HPV16+ cervical intraepithelial neoplasia 2/3 (CIN2/3). USA	Cervical cancer. Phase I/II	Open 2009-ND	HPV16 E7 oncogene	Naked plasmid DNA/intramuscular	*In vivo *
US-1040	Phase I, open-label study to evaluate the safety, tolerability, and immunogenicity of a fourth dose of human papillomavirus (HPV) DNA plasmid (VGX-3100) + electroporation (EP) in adult females previously immunized with VGX-3100. USA	Cervical cancer. Phase I	Open 2010-ND	(i) HPV16 E6 and E7 oncogenes (ii) HPV18 E6 and E7 oncogenes	Naked plasmid DNA/intramuscular	*In vivo *
US-1082	A phase II evaluation of ADXS11-001 (NSC #752718, IND # 13,712) in the treatment of persistent or recurrent squamous or on-squamous cell carcinoma of the cervix. USA	Cervical cancer. Phase II	Open 2010-ND	HPV E7 oncogene	Listeria monocytogenes/intravenous	*In vivo *
US-1093	Phase II placebo-controlled study of VGX-3100, (HPV16 E6/E7, HPV18 E6/E7 DNA Vaccine) delivered IM followed by electroporation (Ep) with cellectra-5p for the treatment of biopsy-proven CIN 2/3 or CIN 3 with documented HPV 16 or 18. USA	Cervical cancer. Phase II	Open 2011-ND	(i) HPV16 E6-E7 fusion protein (ii) HPV18 E6-E7 fusion protein	Naked plasmid DNA/intramuscular	*In vivo *

Clinical trial information obtained from http://www.abedia.com/wiley/index.html.

ND: no data provided.

Note. The table was created by the authors of this paper with the information obtained from http://www.abedia.com/wiley/index.html.

## References

[1] WHO/ICO Information Centre on HPV (2010). Human papillomavirus and related cancers in world. *Summary Report*.

[2] Doorbar J (2006). Molecular biology of human papillomavirus infection and cervical cancer. *Clinical Science*.

[3] Pyeon D, Pearce SM, Lank SM, Ahlquist P, Lambert PF (2009). Establishment of human papillomavirus infection requires cell cycle progression. *PLoS Pathogens*.

[4] Sankaranarayanan R, Budukh AM, Rajkumar R (2001). Effective screening programmes for cervical cancer in low- and middle-income developing countries. *Bulletin of the World Health Organization*.

[5] Schiffman M, Wentzensen N, Wacholder S, Kinney W, Gage JC, Castle PE (2011). Human papillomavirus testing in the prevention of cervical cancer. *Journal of the National Cancer Institute*.

[6] de Villiers EM, Fauquet C, Broker TR, Bernard HU, zur Hausen H (2004). Classification of papillomaviruses. *Virology*.

[7] Bernard HU (2002). Gene expression of genital human papillomaviruses and considerations on potential antiviral approaches. *Antiviral Therapy*.

[8] Zheng Z, Baker C (2006). Papillomavirus genome structure, expression and post-transcription regulation. *NIH Public Access*.

[9] Egawa N, Nakahara T, Ohno S (2012). The E1 protein of human papillomavirus type 16 is dispensable for maintenance replication of the viral genome. *Journal of Virology*.

[10] Ramírez-Salazar E, Centeno F, Nieto K, Valencia-Hernández A, Salcedo M, Garrido E (2011). HPV16 E2 could act as down-regulator in cellular genes implicated in apoptosis, proliferation and cell differentiation. *Virology Journal*.

[11] Horner SM, DiMaio D (2007). The DNA binding domain of a papillomavirus E2 protein programs a chimeric nuclease to cleave integrated human papillomavirus DNA in HeLa cervical carcinoma cells. *Journal of Virology*.

[12] You J (2010). Papillomavirus interaction with cellular chromatin. *Biochimica et Biophysica Acta*.

[13] Centeno F, Ramírez-Salazar E, García-Villa E, Gariglio P, Garrido E (2008). TAF1 interacts with and modulates human papillomavirus 16 E2-dependent transcriptional regulation. *Intervirology*.

[14] Wang Q, Griffin H, Souther S (2004). Functional analysis of the human papillomavirus type 16 E1^∧^E4 protein provides a mechanism for in vivo and in vitro keratin filament reorganization. *Journal of Virology*.

[15] Khan J, Davy CE, McIntosh PB (2011). Role of calpain in the formation of human papillomavirus type 16 amyloid fibers and reorganization of the keratin network. *Journal of Virology*.

[16] Venuti A, Paolini F, Nasir L (2011). Papillomavirus E5: the smallest oncoprotein with many functions. *Molecular Cancer*.

[17] Cortese MS, Ashrafi GH, Campo MS (2010). All 4 di-leucine motifs in the first hydrophobic domain of the E5 oncoprotein of human papillomavirus type 16 are essential for surface MHC class I downregulation activity and E5 endomembrane localization. *International Journal of Cancer*.

[18] Bouvard V, Matlashewski G, Gu ZM, Storey A, Banks L (1994). The human papillomavirus type 16 E5 gene cooperates with the E7 gene to stimulate proliferation of primary cells and increases viral gene expression. *Virology*.

[19] Ghai J, Ostrow RS, Tolar J (1996). The E5 gene product of rhesus papillomavirus is an activator of endogenous Ras and phosphatidylinositol-3′-kinase in NIH 3T3 cells. *Proceedings of the National Academy of Sciences of the United States of America*.

[20] Wise-Draper TM, Wells SI (2008). Papillomavirus E6 and E7 proteins and their cellular targets. *Frontiers in Bioscience*.

[21] Vousden KH, Lane DP (2007). p53 in health and disease. *Nature Reviews Molecular Cell Biology*.

[22] Dick FA, Dyson NJ (2002). Three regions of the pRB pocket domain affect its inactivation by human papillomavirus E7 proteins. *Journal of Virology*.

[23] Moody CA, Laimins LA (2010). Human papillomavirus oncoproteins: pathways to transformation. *Nature Reviews Cancer*.

[24] Alp-Avci G (2012). Genomic organization and proteins of human papillomavirus. *Mikrobiyoloji Bülteni*.

[25] Melsheimer P, Kaul S, Dobeck S, Bastert G (2003). Immunocytochemical detection of HPV high-risk type L1 capsid proteins in LSIL and HSIL as compared with detection of HPV L1 DNA. *Acta Cytologica*.

[26] Toro M, Llombart-Bosch A (2005). Immunohistochemical detection of the high risk Human Papillomavirus (HPV)-L1 protein in uterine cervix cytologies and biopsies. *Revista Española de Patología*.

[27] Molano M, Posso H, Weiderpass E (2002). Prevalence and determinants of HPV infection among Colombian women with normal cytology. *British Journal of Cancer*.

[28] Roteli-Martins CM, de Carvalho NS, Naud P (2011). Prevalence of human papillomavirus infection and associated risk factors in young women in Brazil, Canada, and the United States: a multicenter cross-sectional study. *International Journal of Gynecological Pathology*.

[29] Romanczuk H, Howley PM (1992). Disruption of either the E1 or the E2 regulatory gene of human papillomavirus type 16 increases viral immortalization capacity. *Proceedings of the National Academy of Sciences of the United States of America*.

[30] Trottier H, Burchell AN (2009). Epidemiology of mucosal human papillomavirus infection and associated diseases. *Public Health Genomics*.

[31] Münger K, Baldwin A, Edwards KM (2004). Mechanisms of human papillomavirus-induced oncogenesis. *Journal of Virology*.

[32] Manavi M, Hudelist G, Fink-Retter A, Gschwantler-Kaulich D, Pischinger K, Czerwenka K (2008). Human papillomavirus DNA integration and messenger RNA transcription in cervical low- and high-risk squamous intraepithelial lesions in Austrian women. *International Journal of Gynecological Cancer*.

[33] American Congress of Obstetricians (2012). *New Cervical Cancer Screening Recommendations from the U.S*.

[34] Nanda K, McCrory DC, Myers ER (2000). Accuracy of the papanicolaou test in screening for and follow-up of cervical cytologic abnormalities: a systematic review. *Annals of Internal Medicine*.

[35] Franco EL, Duarte-Franco E, Ferenczy A (2003). Prospects for controlling cervical cancer at the turn of the century. *Salud Publica de Mexico*.

[36] de Cremoux P, Coste J, Sastre-Garau X (2003). Efficiency of the hybrid capture 2 HPV DNA test in cervical cancer screening: a study by the French society of clinical cytology. *American Journal of Clinical Pathology*.

[37] Arbyn M, Buntinx F, van Ranst M, Paraskevaidis E, Martin-Hirsch P, Dillner J (2004). Virologic versus cytologic triage of women with equivocal pap smears: a meta-analysis of the accuracy to detect high-grade intraepithelial neoplasia. *Journal of the National Cancer Institute*.

[38] Cuzick J, Mayrand MH, Ronco G, Snijders P, Wardle J (2006). Chapter 10: new dimensions in cervical cancer screening. *Vaccine*.

[39] Qu W, Jiang G, Cruz Y (1997). PCR detection of human papillomavirus: comparison between MY09/MY11 and GP5+/GP6+ primer systems. *Journal of Clinical Microbiology*.

[40] Strauss S, Jordens JZ, McBride D (1999). Detection and typing of human papillomavirus DNA in paired urine and cervical scrapes. *European Journal of Epidemiology*.

[41] Giovannelli L, Lama A, Capra G, Giordano V, Aricò P, Ammatuna P (2004). Detection of human papillomavirus DNA in cervical samples: analysis of the new PGMY-PCR compared to the hybrid capture II and MY-PCR assays and a two-step nested PCR assay. *Journal of Clinical Microbiology*.

[42] Kleter B, van Doorn LJ, ter Schegget J (1998). Novel short-fragment PCR assay for highly sensitive broad-spectrum detection of anogenital human papillomaviruses. *American Journal of Pathology*.

[43] Josefsson A, Livak K, Gyllensten U (1999). Detection and quantitation of human papillomavirus by using the fluorescent 5′ exonuclease assay. *Journal of Clinical Microbiology*.

[44] Moberg M, Gustavsson I, Gyllensten U (2003). Real-time pcr-based system for simultaneous quantification of human papillomavirus types associated with high risk of cervical cancer. *Journal of Clinical Microbiology*.

[45] Gharizadeh B, Ghaderi M, Donnelly D, Amini B, Wallin KL, Nyrén P (2003). Multiple-primer DNA sequencing method. *Electrophoresis*.

[46] Fontaine V, Mascaux C, Weyn C (2007). Evaluation of combined general primer-mediated PCR sequencing and type-specific PCR strategies for determination of human papillomavirus genotypes in cervical cell specimens. *Journal of Clinical Microbiology*.

[47] Baleriola C, Millar D, Melki J (2008). Comparison of a novel HPV test with the Hybrid Capture II (hcII) and a reference PCR method shows high specificity and positive predictive value for 13 high-risk human papillomavirus infections. *Journal of Clinical Virology*.

[48] An HJ, Cho NH, Lee SY (2003). Correlation of cervical carcinoma and precancerous lesions with human papillomavirus (HPV) genotypes detected with the HPV DNA chip microarray method. *Cancer*.

[49] Kim CJ, Jeong JK, Park M (2003). HPV oligonucleotide microarray-based detection of HPV genotypes in cervical neoplastic lesions. *Gynecologic Oncology*.

[50] Kotaniemi-Talonen L, Nieminen P, Anttila A, Hakama M (2005). Routine cervical screening with primary HPV testing and cytology triage protocol in a randomised setting. *British Journal of Cancer*.

[51] Castle PE, Stoler MH, Wright TC, Sharma A, Wright TL, Behrens CM (2011). Performance of carcinogenic human papillomavirus (HPV) testing and HPV16 or HPV18 genotyping for cervical cancer screening of women aged 25 years and older: a subanalysis of the ATHENA study. *The Lancet Oncology*.

[52] de Sanjose S, Quint WG, Alemany L (2010). Human papillomavirus genotype attribution in invasive cervical cancer: a retrospective cross-sectional worldwide study. *The Lancet Oncology*.

[53] Rijkaart DC, Berkhof J, Rozendaal L (2012). Human papillomavirus testing for the detection of high-grade cervical intraepithelial neoplasia and cancer: final results of the POBASCAM randomised controlled trial. *The Lancet Oncology*.

[54] Ronco G, Giorgi-Rossi P, Carozzi F (2010). Efficacy of human papillomavirus testing for the detection of invasive cervical cancers and cervical intraepithelial neoplasia: a randomised controlled trial. *The Lancet Oncology*.

[55] Katki HA, Kinney WK, Fetterman B (2011). Cervical cancer risk for women undergoing concurrent testing for human papillomavirus and cervical cytology: a population-based study in routine clinical practice. *The Lancet Oncology*.

[56] Murillo R, Wiesner C, Cendales R, Piñeros M, Tovar S (2011). Comprehensive evaluation of cervical cancer screening programs: the case of Colombia. *Salud Pública de México*.

[57] Andrés-Gamboa O, Chicaíza L, García-Molina M (2008). Cost-effectiveness of conventional cytology and HPV DNA testing for cervical cancer screening in Colombia. *Salud Pública de México*.

[58] Vijayaraghavan A, Efrusy M, Lindeque G, Dreyer G, Santas C (2009). Cost effectiveness of high-risk HPV DNA testing for cervical cancer screening in South Africa. *Gynecologic Oncology*.

[59] Qiao YL, Sellors JW, Eder PS (2008). A new HPV-DNA test for cervical-cancer screening in developing regions: a cross-sectional study of clinical accuracy in rural China. *The Lancet Oncology*.

[60] Wright TC, Massad LS, Dunton CJ, Spitzer M, Wilkinson EJ, Solomon D (2007). 2006 consensus guidelines for the management of women with abnormal cervical cancer screening tests. 2006 American Society for Colposcopy and Cervical Pathology-sponsored Consensus Conference. *American Journal of Obstetrics and Gynecology*.

[61] Schlecht NF, Platt RW, Duarte-Franco E (2003). Human papillomavirus infection and time to progression and regression of cervical intraepithelial neoplasia. *Journal of the National Cancer Institute*.

[62] Moscicki AB, Shiboski S, Hills NK (2004). Regression of low-grade squamous intra-epithelial lesions in young women. *Lancet*.

[63] Khan MJ, Castle PE, Lorincz AT (2005). The elevated 10-year risk of cervical precancer and cancer in women with human papillomavirus (HPV) type 16 or 18 and the possible utility of type-specific HPV testing in clinical practice. *Journal of the National Cancer Institute*.

[64] Carreon JD, Sherman ME, Guillén D (2007). CIN2 is a much less reproducible and less valid diagnosis than CIN3: results from a histological review of population-based cervical samples. *International Journal of Gynecological Pathology*.

[65] Mathevet P, Dargent D, Roy M, Beau G (1994). A randomized prospective study comparing three techniques of conization: cold knife, laser, and LEEP. *Gynecologic Oncology*.

[66] Kyrgiou M, Koliopoulos G, Martin-Hirsch P, Arbyn M, Prendiville W, Paraskevaidis E (2006). Obstetric outcomes after conservative treatment for intraepithelial or early invasive cervical lesions: systematic review and meta-analysis. *Lancet*.

[67] Arbyn M, Kyrgiou M, Simoens C (2008). Perinatal mortality and other severe adverse pregnancy outcomes associated with treatment of cervical intraepithelial neoplasia: meta-analysis. *British Medical Journal*.

[68] Nuovo J, Melnikow J, Willan AR, Chan BKS (2000). Treatment outcomes for squamous intraepithelial lesions. *International Journal of Gynecology and Obstetrics*.

[69] Melnikow J, McGahan C, Sawaya GF, Ehlen T, Coldman A (2009). Cervical intraepithelial neoplasia outcomes after treatment: long-term follow-up from the British Columbia Cohort Study. *Journal of the National Cancer Institute*.

[70] Paraskevaidis E, Arbyn M, Sotiriadis A (2004). The role of HPV DNA testing in the follow-up period after treatment for CIN: a systematic review of the literature. *Cancer Treatment Reviews*.

[71] Jones J, Saleem A, Rai N (2011). Human Papillomavirus genotype testing combined with cytology as a ‘test of cure’ post treatment: the importance of a persistent viral infection. *Journal of Clinical Virology*.

[72] Venturoli S, Ambretti S, Cricca M (2008). Correlation of high-risk human papillomavirus genotypes persistence and risk of residual or recurrent cervical disease after surgical treatment. *Journal of Medical Virology*.

[73] ABEDIA http://www.abedia.com/wiley/index.html.

[74] US National Institutes of Health http://www.nlm.nih.gov/copyright.html.

[75] Niu XY, Peng ZL, Duan WQ, Wang H, Wang P (2006). Inhibition of HPV 16 E6 oncogene expression by RNA interference in vitro and in vivo. *International Journal of Gynecological Cancer*.

[76] Salazar-León J, Reyes-Román F, Meneses-Acosta A (2011). Silencing of HPV16 E6 and E7 oncogenic activities by small interference RNA induces autophagy and apoptosis in human cervical cancer cells. *Journal of Nucleic Acids Investigation*.

[77] Jiang M, Milner J (2002). Selective silencing of viral gene expression in HPV-positive human cervical carcinoma cells treated with siRNA, a primer of RNA interference. *Oncogene*.

[78] Chang JTC, Kuo TF, Chen YJ (2010). Highly potent and specific siRNAs against E6 or E7 genes of HPV16-or HPV18-infected cervical cancers. *Cancer Gene Therapy*.

[79] Fujii T, Saito M, Iwasaki E (2006). Intratumor injection of small interfering RNA-targeting human papillomavirus 18 E6 and E7 successfully inhibits the growth of cervical cancer. *International Journal of Oncology*.

[80] Wu SY, Singhania A, Burgess M (2011). Systemic delivery of E6/7 siRNA using novel lipidic particles and its application with cisplatin in cervical cancer mouse models. *Gene Therapy*.

[81] Chesson HW, Blandford JM, Gift TL, Tao G, Irwin KL (2004). The estimated direct medical cost of sexuality transmitted diseases among American youth, 2000. *Perspectives on Sexual and Reproductive Health*.

[82] Blumenthal D (2012). Performance improvement in health care—seizing the moment. *The New England Journal of Medicine*.

[83] Aguilar-Pérez JA, Leyva-López AG, Angulo-Nájera D, Salinas A, Lazcano-Ponce EC (2003). Tamizaje en cáncer cervical: conocimiento de la utilidad y uso de citología cervical en México. *Revista de Saúde Pública*.

[84] Madrigal-Campa MA, Lazcano-Ponce ED, Infante-Catañeda C (2005). Sobreutilización del servicio de colposcopia en México. *Ginecología y Obstetricia de México*.

[85] Mahdavi A, Monk BJ (2005). Vaccines against human papillomavirus and cervical cancer: promises and challenges. *Oncologist*.

